# A Superior Spermatic Cord Denervation Technique?: A Meta‐Analysis

**DOI:** 10.1111/andr.70422

**Published:** 2026-11-09

**Authors:** Daniel Turin, Lane Shish, Ryn Gagen, Logan Hubbard, Joshua Bodie

**Affiliations:** ^1^ School of Medicine University of Minnesota Minneapolis Minnesota USA; ^2^ Department of Urology University of Minnesota Minneapolis Minnesota USA; ^3^ Health Sciences Library University of Minnesota Minneapolis Minnesota USA

**Keywords:** denervation, orchialgia, spermatic cord, spermatic cord denervation

## Abstract

**Introduction:**

Chronic orchialgia is a common condition encompassing about 2.5%–5% of urology consults in the United States each year. When conservative treatments fail, spermatic cord denervation is often pursued after a diagnostic cord block. A standard total denervation (SD) of the spermatic cord has been considered the gold standard, targeting all nerves while preserving arteries. Recently, newer methods like targeted complex denervation (TD) and robotic‐assisted denervation (RD) have emerged.

**Objectives:**

This study systematically evaluates whether these methods provide improved pain relief over the traditional approach.

**Methods:**

A systematic review evaluating pain improvement in men with chronic orchialgia following SD, TD, and RD was conducted, PROSPERO database (Registration No.: CRD42024540544). A comprehensive literature search was performed using MEDLINE, EMBASE, Cochrane Library, Scopus, ClinicalTrials.gov, WHO ICTRP, Global Index Medicus, and Web of Science in May 2024. Articles were evaluated by two independent screeners. Data was analyzed and summarized using odds ratios (ORs) and confidence intervals (CIs) for complete (100%) or significant (> 50%) pain resolution, and continuous variables were compared using inverse‐variance weighted means and corresponding 95% CI. Study quality was assessed using the Newcastle–Ottawa Scale (NOS).

**Results:**

Twenty‐three studies were included: 17 on SD, 5 on RD, and 4 on TD. SD was performed on 675 testicular units, RD on 947, and TD on 992. Complete pain resolution was found in 65.8%, 48.6%, and 51.4% of testicular units undergoing SD, TD, and RD, respectively. Significant (> 50%) pain resolution was experienced by 84.9%, 83.8%, and 83.3% of testicular units after SD, TD, and RD, respectively. SD had 1.86‐fold higher odds of complete pain relief (OR: 1.86; 95% CI: 1.51–2.29; *p* < 0.001) and no difference in the odds of significant (> 50%) pain relief (OR: 0.94; 95% CI: 0.72–1.24; *p* = 0.68) compared to TD. SD had 1.68‐fold higher odds of complete pain relief (OR: 1.68; 95% CI: 1.36–2.07; *p* < 0.001) and no difference in the odds of significant (> 50%) pain relief (OR: 0.98; 95% CI: 0.74–1.28; *p* = 0.86) when compared to RD. Average operative times were 71.50, 20.89, and 21.25 min per testicular unit for SD, TD, and RD, respectively. Complication rates were similar with 6.1%, 7.0%, and 7.6% for standard, targeted, and robotic approaches, respectively. Of all included and scored studies, 55% (12/22) were categorized as having a NOS score < 7.

**Conclusion:**

SD offers the highest odds of complete pain resolution, although all approaches have similar chances of achieving > 50% pain reduction. The ORs suggest that SD had higher odds of complete pain resolution, but the lack of well‐designed comparative studies limits adequate comparison of efficacy.

## 1 Introduction

Chronic orchialgia is a common, debilitating condition that accounts for about 2.5%–5% of urology consultations in the United States each year [1]. It is defined as testicular pain for at least 3 months which interferes with the daily activities and quality of life [2]. This condition can be caused directly such as from prior vasectomy, infection, varicocele, or scrotal trauma [2, 3]. However, it can also be attributed to referred abdominal pain, radiculopathies, or be idiopathic [2, 3]. Conservative management, including heat/ice, analgesics, tricyclic antidepressants, anticonvulsants, and pelvic floor physical therapy (PFPT), is typically the first‐line treatment. However, the success of these methods is often limited [3]. If conservative methods fail, often the next step is to perform a spermatic cord block. If the block improves symptoms by 50%, then surgical intervention with spermatic cord denervation is a consideration [2, 3]. Spermatic cord denervation is shown to be quite successful for those who respond to a spermatic cord block. In one example, 88% of men who had complete pain relief with spermatic cord block had complete postoperative pain relief [3]. Historically, the gold standard method of choice had been the standard denervation (SD) of the spermatic cord which is performed under an operative microscope where the arteries and some lymphatics are preserved while ligating the remaining spermatic cord structures, including nerves and possibly the vas deferens [4]. Recently, different techniques like robotic denervation (RD) and targeted denervation (TD) have emerged, but their benefit over the traditional approach has not been systematically evaluated. TD is based on identification of the trifecta nerve complex, an anatomical area within the spermatic cord that exhibits higher rates of Wallerian degeneration in patients with chronic orchialgia [5]. These three areas are the posterior part of the cremasteric muscle layer, perivasal tissues, and posterior periarterial/lipomatous tissue [5]. TD targets these specific regions for treatment and it has been shown to be a faster operative procedure when compared to SD [4]. This study seeks to systematically evaluate whether RD or TD provides superior pain relief compared to the SD approach.

## 2 Methods (Evidence Acquisition)

### 2.1 Search Strategy

This systematic review and meta‐analysis was conducted in accordance with the Preferred Reporting Items for Systematic Reviews and Meta‐Analyses (PRISMA) guidelines [6]. The protocol was pre‐registered in the PROSPERO database (Registration No.: CRD42024540544). A comprehensive literature search was performed using MEDLINE, EMBASE, Cochrane Library, Scopus, ClinicalTrials.gov, WHO ICTRP, Global Index Medicus, and Web of Science from January 1st, 1946 to May 15th, 2024. The search strategy incorporated Medical Subject Headings (MeSH) terms and keywords including “scrotal pain,” “chronic scrotal pain,” “acute scrotum,” “orchialgia,” “orchiodynia,” or “testalgia.” The full search strategy is detailed in Supporting Information S1. Reference lists of included studies were also evaluated for potentially eligible studies that were not identified by the search above.

### 2.2 Selection Process

Two reviewers (L.S. and D.T.) independently screened and assessed the eligibility of identified publications using the Covidence data management tool [7]. Publications were included if they assessed patient pain improvement after targeted spermatic cord denervation, robotic spermatic cord denervation, or complete spermatic cord denervation in men ≥ to 18 years old with scrotal/testicular pain for 3 months or more. Randomized controlled trials (RCTs), cohort studies, case series, and reports were included if they reported number or percentage of patients with complete (100%) or significant (> 50%) resolution of pain at the end of the study as reported by questionnaire, verbal report, Visual Analog Scale (VAS), Personal Insight Question 6 (PIQ‐6) tool, or other tool defined to score pain by the study. Publications were excluded if they included men with acute orchialgia, were reviews, letters, editorials, applications on animal, or ex vivo models, only included preliminary data, or studies describing technical aspects of the surgery but omitting outcomes related to pain.

Our primary outcomes of interest were the degree of pain resolution looking at the percentage of patients in each group with complete (100%) pain relief and significant (> 50%) pain relief for each surgical intervention. These pain score measures could vary as determined by the questionnaire unique to each study using verbal reporting, VAS score, PIQ‐6, or other tools used by the study. Second, outcomes include surgical complications, procedure time, reoperation rate, and expense of the operation.

To assure inter‐rater reliability, screening was calibrated with a random 10% selection of the citations. The two independent screeners then evaluated the appropriateness of including these citations in the study based on the inclusion/exclusion criteria. The inter‐rater reliability was then performed by using Cohen's kappa value. A Cohen's kappa value of 0.8–1 needed to be obtained to identify strong agreement between the screeners. If Cohen's kappa value was not 0.8–1 (at least 80% agreement), consensus on conflicting decisions needed to be reached through discussion, and then the screening calibration process would need to be repeated. If no consensus was met, then a third screener (J.B.) would be the tie‐breaker.

Next, a first round of screening was performed by the two independent screeners (L.S. and D.T.) which evaluated only the title/abstracts of the captured citations and determined if they should be included in the next round of screening based on the inclusion/exclusion criteria. If it was unclear, then the record was included for the screening of the full text in the next round. Afterward, a second screening commenced of the full‐text documents included from the first round, which were again screened by the same screeners.

### 2.3 Data Collection

Data extraction was performed in duplicate by two evaluators (L.S. and D.T.). The data were compared for correctness and disagreements were resolved with consensus. The data were then stored in a data collection tool which organized the information. Data extracted included percentage/number of patients with complete (100%) pain resolution after the intervention, percentage/number of patients with significant (> 50%) pain resolution mean operative time (averaged for one cord unit), mean estimated cost, complications experienced, reoperation rates, type of procedure performed (SD, TD, or RD), duration of follow‐up, and patient demographics. For data that were unclear, missing, and appeared to be duplicate, we contacted the respective corresponding authors for clarification. Available data were included while unavailable elements were recorded as “not provided.” If authors of suspected duplicate datasets could not be reached, two reviewers assessed overlap (outcomes, study size, time period) to determine inclusion.

### 2.4 Quality Assessment

The risk of bias (RoB) for included studies was assessed using the Newcastle–Ottawa Scale (NOS) for nonrandomized studies [8]. Each study was categorized as having a low, moderate, or high RoB. The reported assessments are shown in Table 1. Disagreements were settled by consensus. To evaluate for publication bias, an Egger's test was performed for each approach.

**TABLE 1:** Newcastle–Ottawa Scale evaluation of included studies.

	Selection				Comparability	Outcome			Total
	1	2	3	4	1	1	2	3	
de Oliveira et al. [13]	★	★	★	★	—	★	★	★	7
Ahmed et al. [14]	—	★	★	★	—	—	—	★	4
Cassidy [15]	★	★	★	★	—	★	★	★	7
Heidenreich et al. [16]	★	★	★	★	—	—	★	★	6
Tan et al. [17]	★	★	★	★	—	★	★	★	7
Murthy et al. [18]	★	★	★	★	—	★	—	—	5
Shergill et al. [19]	★	★	★	★	—	—	★	★	6
Tu et al. [12]	*Not scored – case report*								
Beamer et al. [20]	★	★	★	★	—	★	★	—	6
Oomen et al. [21]	★	★	★	★	—	★	★	★	7
Strom and Levine [22]	★	★	★	★	—	★	★	★	7
Marconi et al. [23]	★	★	★	★	—	★	★	★	7
Dover and Williams [24]	—	★	★	★	—	—	★	★	5
Chaudhari et al. [25]	★	★	★	★	—	★	★	★	7
Raheem et al. [26]	★	★	★	★	—	★	—	★	6
Larsen et al. [27]	★	★	★	★	—	★	★	—	6
Kavoussi et al. [28]	★	★	★	★	—	★	★	★	7
Long et al. [29]	★	★	★	★	—	★	★	★	7
Calixte et al. [30]	★	★	★	★	—	★	★	★	7
Etafy et al. [31]	★	★	★	★	—	★	★	—	6
Parekattil et al. [32]	★	★	★	★	—	★	—	—	5
Parekattil et al. [33]	★	★	★	★	—	★	—	—	5
Gudeloglu et al. [34]	★	★	★	★	—	★	★	—	6

### 2.5 Statistical Analysis

All statistical analyses were performed using R (R Foundation for Statistical Computing, Vienna, Austria), GraphPad Prism, and Microsoft Excel [9, 10, 11]. All data collected from primary studies were reported in primary tables or in Supporting Information. Dichotomous outcomes were summarized as frequencies, percentages, or odds ratios (ORs) with 95% confidence intervals (CIs). Continuous outcomes were summarized using inverse‐variance weighted mean differences with corresponding 95% CIs. Each study was weighted by the inverse of the variance of its effect estimate, such that studies with greater precision (smaller variance) contributed proportionally more to the pooled estimate. For the continuous variables such as mean operating room time, the inverse‐variance weighting method was used to calculate the mean of means and 95% CI of each approach.

Because substantial clinical and methodological heterogeneity was anticipated among the included studies, including differences in study design, patient populations, follow‐up duration, pain assessment methods, and surgical technique, pooled estimates were calculated using a random‐effects model with inverse‐variance weighting. This approach incorporates both within‐study sampling error and between‐study variability, providing more conservative estimates than a fixed‐effect model.

Between‐study heterogeneity was evaluated using Cochran's Q statistic and quantified using the *I* ^2^ statistic. Heterogeneity was interpreted according to Cochrane recommendations, with *I* ^2^ values of 0%–40% considered potentially unimportant, 30%–60% moderate, 50%–90% substantial, and 75%–100% considerable heterogeneity. If the calculation was unable to be completed due to a division by zero, a continuity correction was applied by adding 0.5 to the numerator and denominator and the calculation was then performed.

Prespecified subgroup analyses were performed according to study design (prospective vs. retrospective) and methodological quality (NOS score > 6 vs. ≤ 6) to explore potential sources of heterogeneity. Sensitivity analyses were conducted by restricting analyses to prospective studies and separately to studies at lower RoB (NOS score > 6).

Meta‐regression was considered but was not performed because the number of available studies within each treatment subgroup was insufficient to provide reliable estimates.

For comparisons between pooled treatment effects, *z*‐tests were performed using pooled log ORs and their standard errors. No independent sample *t*‐tests were used for pooled meta‐analytic outcomes. Statistical significance was defined as a two‐sided *p* value < 0.05.

## 3 Results

### 3.1 Search Results

The initial search identified 560 studies of which 299 duplicate studies were excluded (Figure 1). The initial screening was performed on 261 study abstracts by the two independent reviewers resulting in 81 full‐text articles being reviewed for inclusion. In total, 58 studies were excluded for the reasons described in Figure 1. Ultimately, 23 studies were included into the data analysis. Characteristics of the included and excluded studies are shown in Tables S1 and 2, respectively. Standard microscopic spermatic cord denervation was performed in 17 studies (16 evaluated only SD and 1 evaluated TD and SD), targeted spermatic cord denervation was performed in 4 studies (1 evaluated only microscopic targeted, 2 evaluated robotic targeted, and 1 evaluated standard and targeted microscopic denervation), and robotic spermatic cord denervation was performed in 5 studies (3 evaluated a robotic spermatic cord denervation and 2 evaluated a robotic targeted denervation). There were 675, 992, and 947 testicular units in the standard, targeted, and robotic spermatic cord denervation groups, respectively.

**FIGURE 1:**
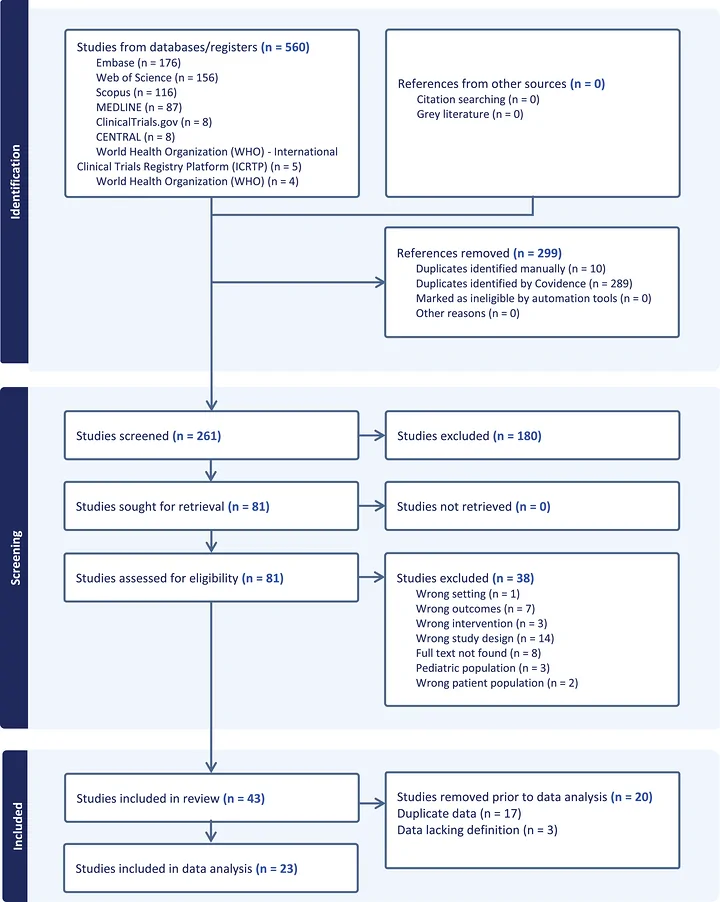
PRISMA flow diagram.

**TABLE 2:** Description of excluded studies.

Excluded full‐text studies			
Study	Journal	Reason for exclusion	Further description for exclusion
Moubasher et al. [61]	Cureus	Wrong study design	Systematic review
Parekattil et al. [62]	Research and Reports in Urology	Wrong study design	Literature review
Vanoli et al. [63]	Revue Medicale Suisse	Wrong study design	Literature review
Zuercher et al. [64]	Canadian Journal of Anesthesia	Pediatric population	—
Goedde et al. [65]	Journal of Osteopathic Medicine	Wrong outcomes	No definitive measurement of pain resolution, the case series focuses on AmnioFix for outcomes
Parekattil [66]	Current Urology Reports	Wrong study design	Literature review
Shiraishi et al. [37]	Urology	Wrong patient population	High inguinal approach to denervation
Tan and Levine [67]	Sexual Medicine Reviews	Wrong study design	Systematic review
Nariculam et al. [68]	BJU International	Wrong outcomes	Focus on spermatic cord block rather than denervation procedure
Levine et al. [69]	Journal of Urology	Wrong outcomes	Follow‐up pain improvement not identified
Devine Jr. and Schellhammer [70]	Transactions of the American Association of Genito‐Urinary Surgeons	Wrong study design	Literature review
DeFreitas et al. [71]	Journal of Sexual Medicine	Wrong study design	Literature review
Nashed et al. [72]	Canadian Urological Association Journal	Wrong outcomes	Compared outcomes from treatments to chronic scrotal content pain. No clear delineation in pain improvement between interventions
Beamer et al. [73]	Canadian Urological Association Journal	Wrong study design	Focus on spermatic cord block rather than denervation procedure
Ergun et al. [38]	AME Medical Journal	Wrong study design	Literature review
Shiraishi and Matsuyama [74]	Journal of Sexual Medicine	Pediatric population	—
Britton et al. [75]	Journal of Sexual Medicine	Full text not found	—
Parekattil et al. [76]	Journal of Urology	Wrong intervention	Literature review
Simon et al. [77]	Journal of Sexual Medicine	Wrong intervention	Focus on spermatic cord block rather than denervation procedure
Parekattil et al. [78]	Journal of Urology	Full text not found	—
Vij et al. [79]	Journal of Urology	Wrong intervention	Mechanism of denervation unclear
Chung et al. [80]	BJU International	Wrong setting	Mechanism of denervation unclear
Shiraishi et al. [81]	Journal of Urology	Pediatric population	—
Basilius et al. [82]	Journal of Sexual Medicine	Wrong outcomes	Quality of life following procedure
Tan et al. [83]	Journal of Sexual Medicine	Wrong patient population	Focus on post vasectomy pain syndrome, no clear definition of chronic orchialgia
Richgels et al. [84]	Journal of Urology	Full text not found	—
Gudeloglu et al. [85]	Journal of Endourology	Wrong outcomes	Review of robotic microsurgery
Larsen and Levine [86]	Journal of Sexual Medicine	Wrong study design	Focus on patients who have previously failed other treatment for chronic scrotal pain then receiving MDSC
Parekattil et al. [87]	Journal of Endourology	Full text not found	—
Witjens et al. [88]	Nederlands Tijdschrift voor Urologie	Wrong outcomes	Focus on spermatic cord block rather than denervation procedure
Witjens et al. [89]	Journal of Urology	Full text not found	—
Parekattil and Cohen [90]	Fertility and Sterility	Full text not found	—
Levine [91]	Journal of Sexual Medicine	Wrong study design	Literature review
Strom and Levine [92]	Current Sexual Health Reports	Wrong study design	No definitive pain measure
Kelsey [93]	Nature Reviews Urology	Wrong study design	Literature review
Brahmbhatt et al. [94]	Robotics in General Surgery	Wrong study design	Literature review
Lorenz [95]	Aktuelle Urologie	Full text not found	—
Levine and Matkov [96]	Journal of Urology	Wrong outcomes	Follow‐up time unclear
El‐Achkar et al. [42]	Andrology	Data unclear if *n*# refers to patients or testicular units	—
El‐Achkar et al. [59]	Journal of Sexual Medicine	Data unclear if *n*# refers to patients or testicular units	—
Hammad et al. [60]	Journal of Sexual Medicine	Data unclear if *n*# refers to patients or testicular units	—
Kavoussi et al. [35]	Asian Journal of Andrology	Duplicate data	Study included under: Kavoussi et al. [28]
Marconi et al. [43]	Journal of Urology	Duplicate data	Study included under: Marconi et al. [23]
Strom and Levine [44]	Journal of Urology	Duplicate data	Study included under: Strom and Levine [22]
Heidenreich et al. [45]	European Urology	Duplicate data	Study included under: Heidenreich et al. [16]
Calixte et al. [46]	Journal of Urology	Duplicate data	Study included under: Calixte et al. [30]
Cassidy [47]	Canadian Urological Association Journal	Duplicate data	Study included under: Cassidy [15]
Parekattil et al. [48]	Journal of Sexual Medicine	Duplicate data	Study included under: Calixte et al. [30]
Tojuola et al. [49]	Journal of Urology	Duplicate data	Study included under: Calixte et al. [30]
Gudeloglu et al. [50]	European Urology	Duplicate data	Study included under: Calixte et al. [30]
Gudeloglu et al. [51]	Journal of Sexual Medicine	Duplicate data	Study included under: Gudeloglu et al. [34]
Gudeloglu et al. [52]	Journal of Sexual Medicine	Duplicate data	Study included under: Gudeloglu et al. [34]
Gudeloglu et al. [53]	Journal of Endourology	Duplicate data	Study included under: Gudeloglu et al. [34]
Gudeloglu et al. [54]	Journal of Endourology	Duplicate data	Study included under: Gudeloglu et al. [34]
Oomen et al. [55]	Urology	Duplicate data	Study included under: Oomen et al. [21]
Parekattil et al. [56]	Journal of Endourology	Duplicate data	Study included under: Calixte et al. [30]
Parekattil et al. [57]	Fertility and Sterility	Duplicate data	Study included under: Calixte et al. [30]
Müller [58]	Aktuelle Urologie	Duplicate data	Study included under: Marconi et al. [23]

### 3.2 Quality Assessment

NOS assessment rating for each study is shown in Table 1. Methodological quality of the 22 included cohort studies was considered “poor” mostly due to a score of 0 in the comparability domain. Of all included and scored studies, 55% (12/22) were categorized as having a NOS score < 7 (Table 1). One case report was not assessed using NOS assessment [12].

### 3.3 Standard Microsurgical Denervation of Spermatic Cord

Pain improvement after SD was provided in 17 of the 23 included studies and evaluated a total of 675 testicular units (Table 3) [12, 13, 14, 15, 16, 17, 18, 19, 20, 21, 22, 23, 24, 25, 26, 27, 28]. The number of testicular units evaluated by each study ranged from 1 to 103. Significant pain reduction (> 50%+ complete resolution) and complete (100%) pain relief were found in 84.9% and 65.8% of testicular units after SD, respectively (Table 3). Of all the testicular units intervened upon, there was a 6.10% complication rate with testicular loss/atrophy, hematoma, and wound infections being the most common complications. A full description of the number and type of complication can be found in Appendix 1 in the Supporting Information. Two studies did not provide information on complications. No study reported a need for repeat spermatic cord denervation due to recurrent pain. Mean study follow‐up ranged from 3 to 52 months. Mean operative time per unit was provided by seven studies and ranged from 39 to 130 min. The mean of means for standard microscopic spermatic cord denervation operating time was 71.50 min for the seven studies providing data on duration (Figure 2). No study provided information on the estimated cost of the operation.

**TABLE 3:** Spermatic cord pain improvement summary by approach.

Denervation analysis					
	Standard denervation	Targeted denervation		Robotic denervation	
Cord units	675	992		947	
Pain improvement: post denervation, *n* (%)					
< 50%	102 (15.11%)	161 (16.23%)		158 (16.68%)	
> 50%	129 (19.11%)	349 (35.18%)		302 (31.89%)	
Complete resolution	444 (65.78%)	482 (48.59%)		487 (51.43%)	
Significant pain reduction (> 50% + complete resolution)	573 (84.89%)	831 (83.77%)		789 (83.33%)	
Risk of pain improvement: Standard denervation vs. intervention, OR (CI)					
> 50% improvement	—	0.94 (0.72–1.24)	*p* = 0.678	0.98 (0.74–1.28)	*p* = 0.860
Complete resolution	—	1.86 (1.51–2.29)	*p* < 0.001	1.68 (1.36–2.07)	*p* < 0.001
Operative time per cord (weighted average)	71.50 min	20.89 min		21.25 min	
Reoperation rate	1.80%	0%		—	
Complication rate	6.10%	7.00%		7.60%	

**FIGURE 2:**
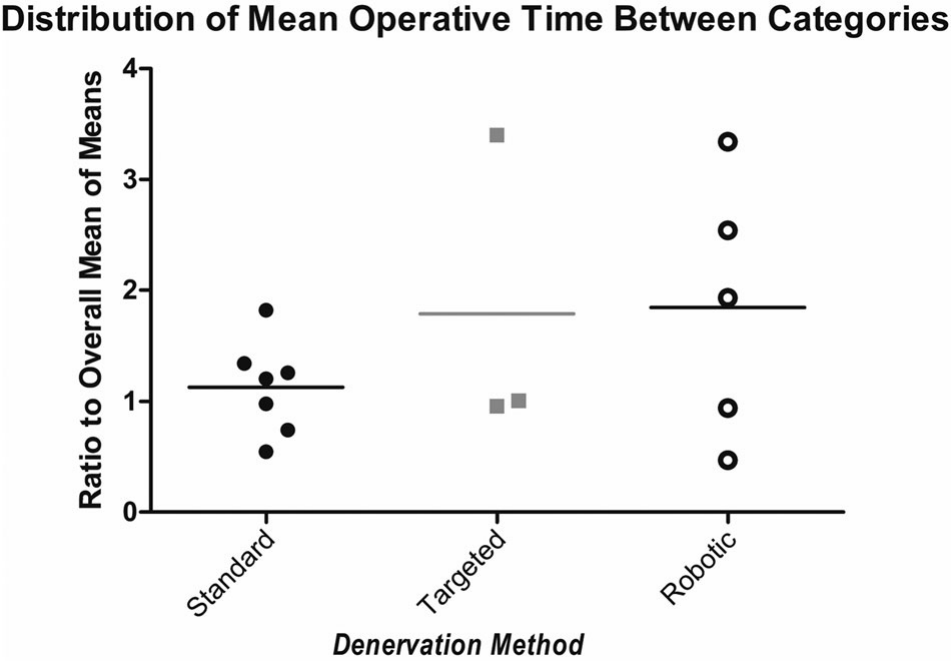
Mean of means for operative time by method.

After standard microscopic spermatic cord denervation, weighted odds of 1.76 (95% CI: 1.49–2.08) for complete pain relief were observed, which was statistically significant (Figure 3). The *I* ^2^ statistic was 89.3%, demonstrating that there may be substantial to considerable heterogeneity between studies. Weighted odds of 4.84 (95% CI: 3.90–6.00) were demonstrated for significant (> 50%) pain relief, which was statistically significant (Figure 4). The *I* ^2^ statistic was 64.6%, demonstrating that substantial heterogeneity was possibly present between studies.

**FIGURE 3:**
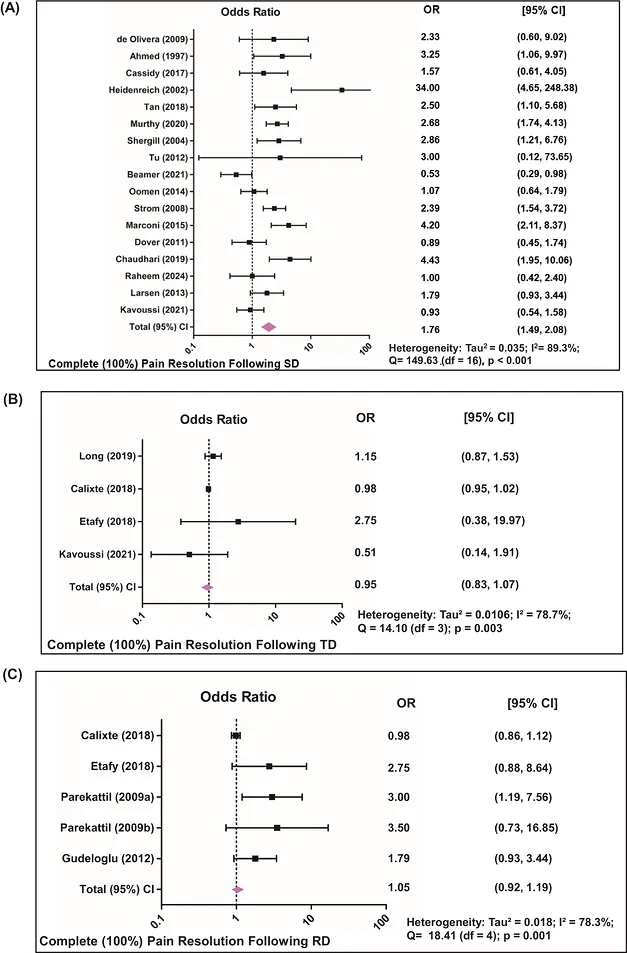
Complete pain resolution by SD (A), TD (B), and RD (C). Forest plots demonstrating odds of complete pain resolution.

**FIGURE 4:**
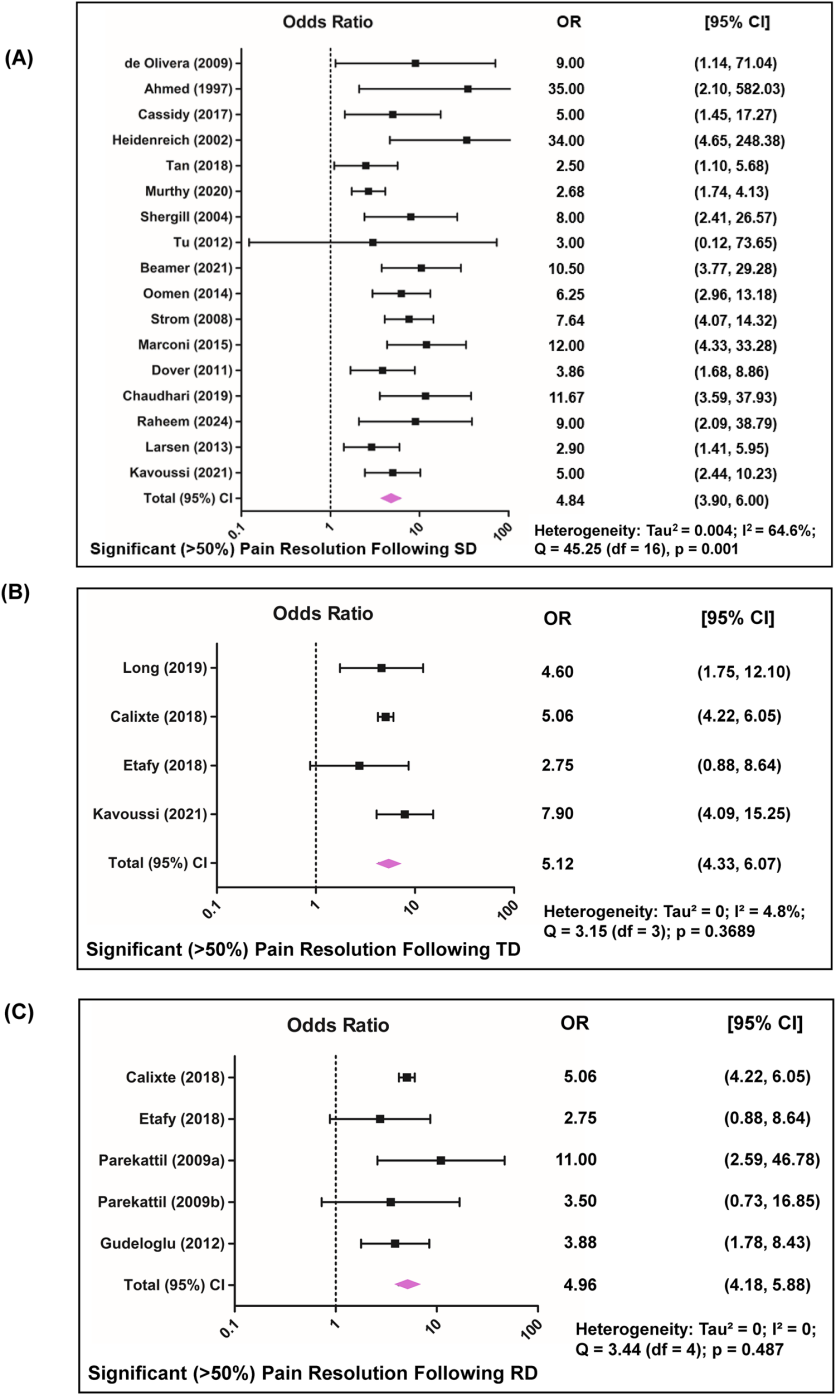
Significant (> 50%) pain resolution by SD (A), TD (B), and RD (C). Forrest plots demonstrating odds of significant pain resolution.

Egger's regression of the complete pain resolution data demonstrated an intercept of −31.3 (*p* < 0.05), which was statistically significant suggesting potential publication bias. For the significant (> 50%) pain resolution data, Egger's regression demonstrated an intercept of −22.1 (*p* = 0.04), which was statistically significant and suggests potential publication bias (Supporting Information S2).

### 3.4 Targeted Microscopic Denervation of Spermatic Cord

TD was evaluated in four studies and evaluated a total of 992 testicular units [28, 29, 30, 31]. The number of testicular units evaluated ranged from 15 to 860. Significant pain reduction (> 50%+ complete resolution) and complete (100%) pain relief were found in 83.8% and 48.6% of testicular units after TD, respectively (Table 3). A complication rate of 7.0% was found with hematoma, wound infection, and wound dehiscence being the most common complications. A full description of the number and type of complication can be found in Appendix 1 in the Supporting Information. Only one study did not provide a description of possible complications. No studies reported the need for repeat spermatic cord denervation due to persistent pain. Mean follow‐up of the studies ranged from 3 to 12 months. Mean operating time per unit was reported by all studies and ranged from 20 to 71 min. The mean of means for operating time using the targeted approach was 20.89 min (Figure 2). No studies provided information on the estimated costs of the operation.

The weighted odds of complete pain resolution after TD were 0.95 (95% CI: 0.83–1.07), which was not statistically significant (Figure 3). The *I* ^2^ statistic was 78.7%, demonstrating possible substantial to considerable heterogeneity between studies. There were statistically significant weighted odds of 5.12 (95% CI: 4.33–6.07) for significant (> 50%) pain resolution after TD (Figure 4). There was minimal evidence of heterogeneity with an *I* ^2^ statistic of 4.79%.

Evaluation of publication bias for complete pain resolution data after a targeted approach with Egger's regression demonstrated an intercept of 1.00 (*p* = 0.92), suggesting no presence of publication bias. Egger's regression for significant (> 50%) pain resolution data demonstrated an intercept of 0.51 (*p* = 0.95), indicating no significant publication bias (Supporting Information S2).

### 3.5 Robotic Denervation of Spermatic Cord

RD was evaluated in five studies which included a total of 947 testicular units [30, 31, 32, 33, 34]. The number of units evaluated per study was between 9 and 860. Significant pain reduction (> 50%+ complete resolution) and complete (100%) pain relief were found in 83.3% and 51.43% of testicular units after RD, respectively (Table 3). A complication rate of 7.60% was found with hematoma, wound infection, and wound dehiscence being the most common complications. A full description of the number and type of complication can be found in Appendix 1 in the Supporting Information. One study did not provide a description of complications. No studies reported the need for repeat spermatic cord denervation due to continued pain. The mean timeframe for follow‐up was 6–12 months. Mean operative time per unit was reported by all but one study and was between 10 and 71 min. The mean of means for operative time using the robotic approach was 21.25 min (Figure 2). No studies provided information on the estimated costs of the operation.

The weighted odds of complete pain resolution after RD were 1.05 (95% CI: 0.92–1.19), which was not statistically significant (Figure 3). The *I* ^2^ statistic was 78.3%, demonstrating potential substantial to considerable heterogeneity between studies. After RD, statistically significant weighted odds of significant pain resolution were observed: 4.96 (95% CI: 4.18–5.88) (Figure 4). There was no evidence of heterogeneity demonstrated, with an *I* ^2^ statistic of 0% for significant (> 50%) pain resolution.

Evaluation for publication bias for complete pain resolution after a robotic approach with Egger's regression exhibited an intercept of 11.6 (*p* = 0.003), which was statistically significant and suggestive of significant publication bias. Egger's regression for significant (> 50%) pain resolution for a robotic approach was −0.17 (*p* = 0.98), demonstrating no significant publication bias (Supporting Information S2).

### 3.6 SD Compared to TD

When comparing SD to TD, SD had 1.86‐fold higher odds of complete pain relief (OR: 1.86; 95% CI: 1.51–2.29; *p* < 0.001), but no difference in the odds of significant (> 50%) pain relief compared to TD (OR: 0.94; 95% CI: 0.72–1.24; *p* = 0.68) (Figure 5). The complication rate between the two approaches was not statistically different (6.1% for SD vs. 7.0% for TD; *p* = 0.49). The difference in operative times was unable to be statistically compared as the standard error or raw data was unable to be obtained from the included studies.

**FIGURE 5:**
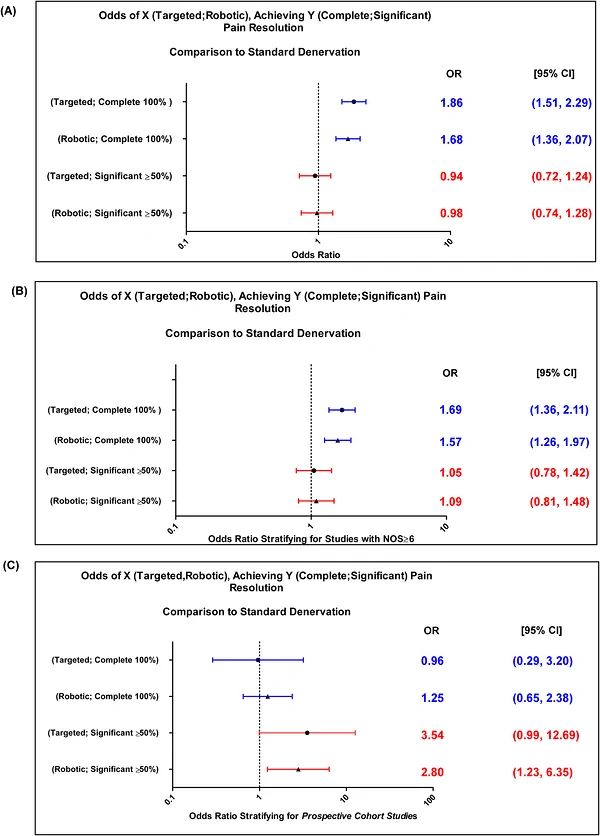
Odds of complete or significant pain relief when comparing SD to TD or RD for all studies (A), only studies with NOS score > 6 (B), and only prospective studies (C).

Subgroup analysis comparing SD to TD utilizing only studies with a prospective study design demonstrated no significant difference in the odds for complete (OR: 0.96; 95% CI: 0.29–3.20; *p* = 0.95) or significant (OR: 3.54; 95% CI: 0.99–12.69; *p* = 0.05) pain relief (Figure 5). In addition, subgroup analysis including only studies with a NOS score > 6 showed an OR of 1.69 in the SD group for increased odds of complete pain relief (OR: 1.69; 95% CI: 1.36–2.11; *p* < 0.001), but no increased odds of significant pain relief (OR: 1.05; 95% CI: 0.78–1.42; *p* = 0.76) compared to TD (Figure 5).

### 3.7 SD Compared to RD

When comparing SD to RD, SD had 1.68‐fold higher odds of complete pain relief (OR: 1.68; 95% CI: 1.36–2.07; *p* < 0.001), but there was no significant difference in the odds of significant (> 50%) pain relief (OR: 0.98; 95% CI: 0.74–1.28; *p* = 0.86) (Figure 5). The complication rates of SD and RD approaches are 6.1% and 7.6%, respectively, and there is no significant difference between the two approaches (*p* = 0.63). Again, the statistical difference in operative times between the two groups was unable to be assessed due to lack of raw data or the standard error of the means provided in the included studies.

Subgroup analysis of the two approaches utilizing only studies with a prospective design demonstrated that SD had no increased odds of complete pain relief (OR: 1.25; 95% CI: 0.65–2.38; *p* = 0.51), but had 2.8 times increased odds of significant (> 50%) pain relief (OR: 2.8; 95% CI: 1.23–6.35; *p* = 0.01) compared to RD (Figure 5). When only including studies with a NOS score > 6, SD had 1.57‐fold improved odds of complete pain relief (OR: 1.57; 95% CI: 1.26–1.97; *p* < 0.001) and no significant difference in significant (> 50%) pain relief (OR: 1.09; 95% CI: 0.81–1.48; *p* = 0.57) compared to RD (Figure 5).

## 4 Discussion

This systematic review and meta‐analysis evaluated the novel surgical approaches of targeted and robotic spermatic cord denervation and compared their effectiveness in pain improvement to standard total denervation for the treatment of chronic orchialgia. The available data showed that the odds for complete pain relief in SD were statistically higher than in TD (Figure 5). While the contributing data are of poor quality, this is logical from an anatomic perspective as the SD completely skeletonizes the spermatic cord and ligates all nerves, while TD only targets nerves in the trifecta, leaving a small number untouched [35]. Though TD could minimize damage to surrounding cord structures by avoiding skeletonization, if affected degenerating nerve elements are left intact, they could contribute to the continued pain following procedure, and could explain why the SD approach demonstrated better odds of complete pain improvement [36]. SD also showed statistically higher odds of complete pain improvement when compared to RD (Figure 5). RD is enticing, as it could give the surgeon a benefit of multiple tools, improved ergonomics, and the potential to eliminate error caused by tremor [36]. However, it is interesting to note that when observing significant (> 50%) pain resolution, there was no difference between approaches (Figure 5). The exact etiology of this result is unclear, but one explanation could be patient selection. Shiraishi et al. describe the importance of psychological evaluation to rule out psychogenic causes of scrotal pain using questionnaires like Beck's Depression Inventory (BDI) [37]. Patients with lower BDI scores were 52% more likely to have complete pain resolution after spermatic cord block compared to those with high scores [37]. By not standardizing psychological evaluation, there is a risk of not ruling out psychogenic causes of pain which may decrease the effectiveness of any surgical intervention.

While the SD approach may increase the odds of complete pain resolution, it appears to come at the cost of longer operative times when compared to TD and RD (Figure 2). While it is not possible to determine if these times are statistically different due to the lack of availability of the studies’ raw data and standard error, these shorter operative times in the TD and RD interventions would allow for surgeons to be more efficient, operate on more patients per day, and possibly decrease postoperative complications like infection [36].

Interestingly, there was no significant difference with regard to the number of complications between interventions. However, one difference in surgical complications between the approaches which stands out is testicular loss/atrophy. In the SD group, there were nine instances of testicular loss/atrophy in comparison to one case from the TD and RD groups (Appendix 1 in the Supporting Information). This is not surprising, given the nature of the SD approach, where the spermatic cord is skeletonized, leaving just lymph channels and blood vessels. The procedure can lend itself to accidentally harming a vital component of the spermatic cord. This outcome was also noted and addressed in the review by Ergun et al., which came to similar conclusions [4]. The SD group also showed a higher proportion of hydroceles (Appendix 1 in the Supporting Information) which again we can attribute to the need for complete skeletonization of the cord and potential damage/ligation of lymph channels [38]. The implication of these findings suggest that a thorough discussion about weighing the benefits in terms of pain control versus risks of testicular atrophy or hydrocele should be had with a patient to determine the appropriate intervention. Overall when comparing the complications between treatments, there was no significant difference between them.

A strength of this systematic review and meta‐analysis is that it is the only known comparative review on spermatic cord denervation approaches: SD, TD, and RD. This review utilized all available literature, adhered to PRISMA guidelines (Appendix 2 in the Supporting Information), and ensured a high quality screening process when determining which studies to include. Finally, this paper compares results from *n* = 2614 testicular units, the largest review to date. This large number of units allows for stronger statistical power and overall better supports the conclusions drawn from these data.

## 5 Limitations

This review, however, is not without limitations. While all included studies described pain relief as “no pain relief” versus “complete pain relief,” some studies used “partial (> 50%) pain relief.” However, one limitation is heterogeneity in the method of pain qualification. The majority of studies used the VAS and PIQ‐6, some studies used Wong–Baker FACES Pain Scale, others used just the question of pain free or not, and others did not give a method of pain measurement. This discrepancy in pain quantification can have a significant impact on the conclusions drawn from the implicated study. While the authors of this review attempted to normalize and match pain ratings, this could create the potential for error. A subsequent limitation to this review is the variability in which subjects are reported in included articles. The majority of included studies conducted analyses using each individual “testicular or scrotal units.” However, Raheem et al. (2024) and Heidenreich et al. (2002) used the number of subjects [16, 26]. To avoid overestimation of effect, authors of this review chose to include each of the 35 and 20 subjects as one testicular unit, respectively. This could have underestimated the effect seen in these studies and introduced potential bias in the analysis.

Another limitation of this systematic review is the methodological quality of included studies. While all observation studies met proper inclusion criteria, several of these studies showed moderate to high ROB as noted by NOS score ≤ 6 (Table 1). Every included study received 0/2 stars for “comparability,” meaning that there was no clear control for confounding variables. This majority of studies with NOS score ≤ 6 could have introduced bias into our overall outcomes for this review. The review also demonstrated heterogeneity between the included studies which may have influenced the results and limited the comparability of evidence. Several pooled analyses demonstrated substantial to considerable statistical heterogeneity (*I* ^2^ > 75%), indicating that variability between study results exceeded that expected by chance alone. Consequently, pooled estimates should be interpreted cautiously, as differences in patient selection, pain assessment instruments, surgical technique, follow‐up duration, and study quality all likely contributed to between‐study variability. Although random‐effects models account for between‐study heterogeneity statistically, they do not eliminate potential clinical heterogeneity or residual confounding.

There was also a concern for bias of the results. To further evaluate the cause of this, studies were stratified by RoB and study design and then compared. When analyzing studies with NOS score > 6, they showed identical findings to the full study comparison (Figure 5). This shows that while there were studies included with higher RoB, their inclusion was not a significant confounding factor. However, when stratifying analysis to only prospective cohort studies, results were noted to be different from the full study comparison (Figure 5). This suggests that the poor study design of the included studies likely introduced bias influencing the results of our review. Future randomized control trials comparing these approaches are needed to confirm these results.

Our study found a statistically significant publication bias in both complete and significant pain resolution in the SD group as well as complete pain resolution in the RD group (Supporting Information S2). This bias could have influenced the findings as studies without significance carry the risk of introducing bias. For our study, this could make the conclusions drawn from the above groups appear more statistically significant than they actually are.

An important consideration in determining the appropriateness of a surgical approach can be the overall cost of the procedure. Though review suggests that SD potentially provides superior complete pain resolution compared to TD and RD in observational studies, it is important to evaluate if this benefit is worth potentially increased costs. Authors completed a speculative brief cost estimate of each study. With respect to SD and TD, there was no literature with the definitive cost of each procedure. However, to calculate the price of “start up,” both of these approaches can be done with a surgical microscope or surgical loupes, which cost $10,000–$25,000+ and $500–$3000, respectively [39]. Childers and Maggard‐Gibbons used financial data from 302 hospitals in California to determine the average cost of OR time was about $36.5/min ($36–$37) [40]. If one were to extrapolate this to the average OR times calculated in this article (mean of means), the OR time for an SD procedure would cost approximately $2609, while TD OR time would cost $765. This extrapolation shows that TD is ∼4 times less expensive than SD in terms of OR time. These cost savings make TD an attractive option, but likely not worth it for the patients who are suffering with chronic pain. Parekattil and Gudeloglu acknowledge that for RD to be feasible, it would need to be performed at a high volume center in order to offset the robotic cost [41]. This cost analysis is exploratory and based on indirect data. Firm cost comparisons between approaches were not possible.

This manuscript highlights the need for future investigation. It would be beneficial to conduct a high quality larger RCT or prospective study directly comparing each approach (i.e., SD vs. RD). Studies included in this paper used different measurements of pain outcomes. It will be important to standardize the way that pain is measured, and ensure that outcomes like “complete” and “> 50% pain improvement” are clearly described. Along with this, it would be interesting to follow patients longer postoperatively in order to better document the durability of pain resolution. Finally, investigating patient characteristics like age or comorbidities (hypertension, diabetes, obesity, etc.) and their response to different surgical approaches might aid in determining how best to treat a patient's chronic orchialgia.

## 6 Conclusion

This is the first systematic review and meta‐analysis comparing the TD and RD of the spermatic cord to the SD approach in the treatment of chronic orchialgia. While operative time was longer, the observational data suggest that the standard approach may achieve higher rates of complete pain relief. Even with heterogeneity between studies, data presented in this manuscript correlate with what has been previously described. However, the results should be viewed cautiously given the exploratory nature of the analysis and high RoB of the included studies, which limits comparison, and conclusions will have a low certainty of evidence. Further high‐quality comparative studies are needed to further define the overall efficacy of each of these surgical approaches.

## Author Contributions

Daniel Turin had full access to all data in the study and takes responsibility for the integrity and accuracy of the data and analysis.

Study concept and design: Daniel Turin, Lane Shish, and Joshua Bodie. Acquisition of data: Daniel Turin, Lane Shish, and Ryn Gagen. Analysis and interpretation of data: Daniel Turin, Lane Shish, and Logan Hubbard. Drafting of the manuscript: Daniel Turin, Lane Shish, and Logan Hubbard.

## Funding

The authors have nothing to report.

## Conflicts of Interest

The authors declare no conflicts of interest.

## Supporting information




**Supporting File 1**: andr70422‐sup‐0001‐SuppMat.pptx

**Supporting File 2**: andr70422‐sup‐0002‐SuppMat.docx

**Supporting File 3**: andr70422‐sup‐0003‐SuppMat.pdf

**Supporting File 4**: andr70422‐sup‐0004‐SuppMat.pptx

**Supporting File 5**: andr70422‐sup‐0005‐TableS1.pptx

## Data Availability

The data supporting the findings of this study are available from the corresponding author upon reasonable request.
