# Supplementary material for: A Superior Spermatic Cord Denervation Technique?: A Meta‐Analysis
Source: Andrology. 2026 Nov 9;15(1):e70422. doi: 10.1111/andr.70422 (PMC13748921; doi:10.1111/andr.70422)
Supplement: Supplementary file 1 [file ANDR-15-0-s004.pdf]

Supplementary Material: S1

Total Microscopic, Targeted, and Robotic Spermatic Cord Denervation: A Systematic Review

Review question:
In men with chronic orchialgia (P), do either the robotic or targeted spermatic cord denervation approach (I) provide significantly different pain reduction (O) compared to the total microscopic approach (C)?

Databases:

1. Medline (via Ovid) - 87
2. Embase (via Ovid) - 176
3. Scopus - 116
4. Web of Science (Core Collection) - 156
5. Cochrane CENTRAL - 8
6. Clinicaltrials.gov - 8
7. WHO ICTRP - 5
8. WHO Global Index Medicus - 4

Total before duplicates removed: 560
Total after duplicates removed: 271

Full Search Strategies:

Medline via Ovid Search Strategy

Note: changes from preliminary search

- Added orchiodynia and testalgia as entry terms / synonyms for orchialgia in Emtree term for scrotal pain
- Truncated pain in line 2 for adjacency due to frequency of "painful" in Emtree entry terms
- Ended up with same number of results as the preliminary search approved by the team (87)

https://login.ezproxy.lib.umn.edu/login?url=http://ovidsp.ovid.com/ovidweb.cgi?T=JS&NEWS=N&PAGE=main&SHAREDSEARCHID=40ss3IrQIPD2rzs3usQ22GNpDbf0DWDp5qijczgxYfkCYsWkOFIru4tUNcJrKkNFD

Ovid MEDLINE(R) ALL <1946 to May 15, 2024>

1 (orchialgia or orchiodynia or testalgia).tw,kf. 184
2 (pain* adj3 (testi* or testes* or scrot* or postvasectom* or post-vasectom*)).tw,kf. 4132
3 1 or 2 4224
4 exp Spermatic Cord/ or spermatic cord*.tw,kf. 4407
5 exp Denervation/ or (de-nerv* or denerv* or neurectom* or microdenerv* or micro-denerv* or MSCD).tw,kf. 97763
6 3 and 4 and 5 **87**

Embase via Ovid Search Strategy

https://login.ezproxy.lib.umn.edu/login?url=http://ovidsp.ovid.com/ovidweb.cgi?T=JS&NEWS=N&PAGE=main&SHAREDSEARCHID=1kpPfsPVXEnagZtMyvdnMQsFUsxblhAR896yZU2aw5f7cb6YoTixsSloqNuHvrDfr

Embase Classic+Embase <1947 to 2024 Week 19>

1 exp scrotal pain/ or exp chronic scrotal pain/ or exp acute scrotum/ 3930
2 (orchialgia or orchiodynia or testalgia).tw,kf. 269
3 (pain* adj3 (testi* or testes* or scrot* or postvasectom* or post-vasectom*)).tw,kf. 6252
4 1 or 2 or 3 8426
5 exp spermatic cord/ or "spermatic cord*".tw,kf. 7814
6 exp denervation/ or (de-nerv* or denerv* or neurectom* or microdenerv* or micro-denerv* or MSCD).tw,kf. 62146
7 4 and 5 and 6 **176**

Scopus Search Strategy

Scopus link 5/15 - **116** results

(INDEXTERMS ("scrotal pain" OR "chronic scrotal pain" OR "acute scrotum") OR TITLE-ABS-KEY (orchialgia OR orchiodynia OR testalgia) OR TITLE-ABS-KEY ((pain*) W/3 (testi* or testes* or scrot* or postvasectom* or post-vasectom*))) AND (INDEXTERMS ("spermatic cord") OR TITLE-ABS-KEY ("spermatic cord*")) AND (INDEXTERMS ("denervation") OR TITLE-ABS-KEY (de-nerv* or denerv* or neurectom* or microdenerv* or micro-denerv* or MSCD))

WoS Search Strategy

WoS link 5/15 - **156** results from Web of Science Core Collection

(TS=(orchialgia OR orchiodynia OR testalgia OR "acute scrotum" OR "scrotal pain") OR TS=((pain*) NEAR/3 (testi* or testes* or scrot* or postvasectom* or post-vasectom*))) AND (TS=("spermatic cord*")) AND (TS=(de-nerv* or denerv* or neurectom* or microdenerv* or micro-denerv* or MSCD))

Cochrane CENTRAL Search Strategy

Cochrane CENTRAL - **8** results

ID Search Hits
#1 orchialgia OR orchiodynia OR testalgia OR "acute scrotum" OR "scrotal pain" 105
#2 pain* near/3 (testi* or testes* or scrot* or postvasectom* or post-vasectom*) 706
#3 #1 OR #2 711
#4 MeSH descriptor: [Spermatic Cord] explode all trees 35
#5 spermatic cord* 146
#6 #4 OR #5 146
#7 MeSH descriptor: [Denervation] explode all trees 6299
#8 de-nerv* or denerv* or neurectom* or microdenerv* or micro-denerv* or MSCD 1717
#9 #7 OR #8 7555
#10 #3 AND #6 AND #9 8

Clinicaltrials.gov

Condition/disease: orchialgia
Results: 8

WHO ICTRP (https://trialsearch.who.int/)

Search: orchialgia
Results: 5

WHO Global Index Medicus

WHO Global Index Medicus link 5/15
Search: (orchialgia OR orchiodynia OR testalgia OR "acute scrotum" OR "scrotal pain") AND (denervation OR denerv* OR neurectom* OR microdenerv* OR micro-denerv* OR MSCD)
Results: 4
